# Dominant Role of Aquaculture Patterns over Seasonal Variations in Controlling Potentially Toxic Elements’ Occurrence and Ecological Risks in Sediments

**DOI:** 10.3390/toxics14010065

**Published:** 2026-01-10

**Authors:** Luna Zhang, Yuyi Yang, Huabao Zheng, Zhi Wang, Weihong Zhang

**Affiliations:** 1 Key Laboratory of Soil Contamination Bioremediation of Zhejiang Province, College of Environmental and Resource Sciences, Zhejiang Agriculture and Forestry University, Hangzhou 311300, China; 15205795350@163.com; 2 State Key Laboratory of Lake and Watershed Science for Water Security, Wuhan Botanical Garden, Chinese Academy of Sciences, Wuhan 430074, China; 3 Hubei Key Laboratory of Wetland Evolution & Ecological Restoration, Wuhan Botanical Garden, Chinese Academy of Sciences, Wuhan 430074, China; 4 Danjiangkou Wetland Ecosystem Field Scientific Observation and Research Station, The Chinese Academy of Sciences & Hubei Province, Wuhan 430074, China; 5 Hubei Key Laboratory of Microbial Transformation and Regulation of Biogenic Elements in the Middle Reaches of the Yangtze River, School of Environmental Ecology and Biological Engineering, Wuhan Institute of Technology, Wuhan 430205, China; yangyy@wit.edu.cn; 6 State Key Laboratory of Green and Efficient Development of Phosphorus Resources, Wuhan Institute of Technology, 206 Guanggu 1st Road, Wuhan 430205, China; 7 Key Laboratory for Environment and Disaster Monitoring and Evaluation of Hubei, Innovation Academy for Precision Measurement Science and Technology, Chinese Academy of Sciences, Wuhan 430077, China; zwang@apm.ac.cn

**Keywords:** aquaculture sediments, potentially toxic elements, rice-crayfish co-culture, ecological risk

## Abstract

Aquaculture faces environmental challenges from sediment contamination by potentially toxic elements. This study investigated how aquaculture patterns and seasons jointly affect the distribution and ecological risks of these potentially toxic elements in sediments. By analyzing and comparing sediment samples from different aquaculture systems across seasons, we found that Mn (mean = 435.42 mg/kg) was the most abundant, followed by Zn (mean = 172.69 mg/kg), Cr (mean = 106.79 mg/kg), and Cu (mean = 63.44 mg/kg). Aquaculture patterns were the primary factor determining the composition of potentially toxic elements, followed by season. Fish farming tended to promote their accumulation in sediments, whereas the rice–crayfish co-culture model effectively reduced the enrichment of potentially toxic elements and their associated ecological risks. Therefore, optimizing aquaculture practices proves more effective in controlling these risks than managing seasonal variations. Moreover, total phosphorus was identified as a key driver of potentially toxic element accumulation in sediments. The results from the rice–crayfish co-culture system indicate that enhanced phosphorus management is crucial for mitigating such risks. Accordingly, it is necessary to develop systematic monitoring and integrated remediation strategies focused on priority metals and their main drivers.

## 1. Introduction

As a crucial global food supply system, aquaculture provides high-quality protein while facing significant challenges from contamination by potentially toxic elements. These metals may enter aquaculture systems through various pathways, such as feed inputs, water sources, and external discharges. Sediments act as a key repository and long-term sink for these contaminants [1]. They can temporarily immobilize potentially toxic elements through adsorption, reducing their bioavailability. However, under changing environmental conditions, sediments may also release these potentially toxic elements, thereby acting as a potential secondary pollution source [2,3]. With the expansion of aquaculture scale and increased intensification levels, potentially toxic element pollution has progressively evolved from single-element to multi-element composite patterns. The accumulation of mercury (Hg), zinc (Zn), arsenic (As), cadmium (Cd), lead (Pb), copper (Cu), nickel (Ni), chromium (Cr), and manganese (Mn) in sediments, along with their potential ecological risks, has emerged as a critical concern threatening aquatic environmental security and aquatic product quality [1].

China accounts for 67% of global aquaculture production, with pond culture being a traditional method in the country, contributing to approximately 74% of its total freshwater aquaculture output [4]. In relatively closed aquaculture systems, potentially toxic elements tend to accumulate in surface sediments through suspended particle settlement, while seasonal fluctuations of environmental factors (e.g., temperature, pH, dissolved oxygen, and microbial activity) may significantly influence their migration and transformation behaviors, leading to dynamic release and redistribution at the sediment–water interface [5]. Specifically, over 99% of potentially toxic elements in natural aquatic environments are associated with sediments [6]. Distinct accumulation-release pathways may emerge due to variations in water exchange rates, feeding management practices, and substrate disturbance levels among different aquaculture patterns, while seasonal climatic variations (e.g., rainfall patterns, temperature shifts, and operational cycles) could further amplify or mitigate contamination risks [1,7,8]. However, systematic studies remain lacking regarding seasonal variation patterns and the driving mechanisms of sediments’ potentially toxic element contents across different aquaculture models, including fish ponds, crayfish ponds, crab ponds, and rice–crayfish co-culture systems.

Elucidating the spatiotemporal differentiation characteristics of multi-metal composite pollution and clarifying the synergistic effects between aquaculture patterns and seasonal factors on potentially toxic element environmental behaviors hold significant scientific value for optimizing aquaculture environmental management, preventing secondary pollution, and ensuring sustainable development of aquatic resources. This study aims to reveal seasonal response patterns of potentially toxic element content in sediments under typical aquaculture patterns through multi-season dynamic monitoring, thereby providing a theoretical basis for precise ecological risk assessment and formulation of differentiated control strategies.

## 2. Materials and Methods

### 2.1. Collection of Sediment Samples

Sediment sampling was collected across four distinct aquaculture systems (fish farming ponds (FF), rice–crayfish co-culture ponds (RS), crayfish-farming ponds (SF), and crab farming ponds (CF)) in the Jianghan Plain during four sampling campaigns conducted in spring (April 2024), summer (July 2024), autumn (October 2024), and winter (January 2025). A total of 160 sediment samples were systematically collected following standardized protocols [9,10]. Sampling locations were strategically selected to avoid hydrological influences from water inlets and drainage outlets, ensuring representation of stable sedimentary environments characteristic of each aquaculture pond (geographical coordinates and site characteristics detailed in Table S1, Supplementary Materials).

The standardized five-point sampling methodology was implemented across all study sites. Within each aquaculture pond, five spatially distributed sampling points were established following a uniform grid pattern. Surface sediments (0–5 cm depth) were collected at each designated site using an acid-washed stainless steel mud scoop. Subsequently, five sub-samples from individual ponds were homogenized to form a representative sample representing each pond, resulting in ten representative samples per aquaculture pattern. Immediately following collection, sediment samples were homogenized, placed in sterile containers, and maintained at 4 °C during transport to the laboratory [9]. This preservation protocol ensured sample integrity prior to analytical procedures for potentially toxic element quantification.

### 2.2. Characterization of Sediment Physicochemical and Nutrients

Nitrogen speciation was determined via cadmium reduction spectrophotometry (EPA Method 353.2) for nitrate (NO_3_^−^-N) and the salicylate–hypochlorite method for ammonium (NH_4_^+^-N), while total nitrogen (TN) and total phosphorus (TP) were quantified using persulfate digestion coupled with UV-Vis detection. Carbon dynamics were assessed through total organic carbon (TOC) analysis via high-temperature combustion (Shimadzu TOC-L) and dissolved organic carbon (DOC) measured after 0.45 μm membrane filtration. Physicochemical properties included pH measured potentiometrically (Thermo Orion 3-Star, Thermo Fisher Scientific, Waltham, MA, USA, ±0.01 precision) and water-soluble nitrogen (WSN) extracted with 2M KCl solution. All analyses followed APHA Standard Methods (23rd Ed.) [11], with method validation ensured through triplicate measurements, procedural blanks, and certified reference materials (NIST 1643e), ensuring data reliability for subsequent ecological interpretations.

### 2.3. Determination of Potentially Toxic Element Content in Sediments

Approximately 0.2 g of each sample (weighed accurately to ±0.0001 g) was placed into a pre-cleaned polytetrafluoroethylene (PTFE) digestion vessel. Then, 4 mL of concentrated nitric acid (HNO_3_, 65%) and 2 mL of hydrofluoric acid (HF, 40%) were added sequentially. Digestion was performed using a microwave digestion system (MDS-8G) to ensure complete decomposition of the silicate matrix. After digestion, the vessels were heated on a temperature-controlled hotplate (140 ± 2 °C) to evaporate the residual acids nearly to dryness. After cooling, the digestates were transferred and diluted to a final volume of 50 mL with ultrapure water (resistivity 18.2 MΩ·cm), followed by filtration through a 0.22 μm nylon membrane.

Analysis was performed using inductively coupled plasma mass spectrometry (ICP-MS, X Series 2, Thermo Fisher Scientific, Bremen, Germany) equipped with a collision/reaction cell (CCT, using helium as collision gas). The target elements included ^66^Zn, ^207^Pb, ^60^Ni, ^55^Mn, ^52^Cr, ^63^Cu, ^75^As, ^111^Cd, and ^200^Hg. Calibration curves were prepared using a multi-element standard solution with appropriate internal standards added to correct for signal drift. The correlation coefficient (R^2^) for each calibration curve exceeded 0.999. To ensure data quality, blank controls and triplicate analyses were included in each batch. The quantification of potentially toxic elements using ICP-MS was based on the work of Rodrigues et al. [12].

### 2.4. Comprehensive Assessment of Potential Ecological Risks

The contamination levels and associated ecological risks of potentially toxic elements in Jianghan Plain aquaculture pond sediments were quantitatively assessed through dual methodological approaches: the geo-accumulation index (I_geo_) and the potential ecological risk index (PERI). 

#### 2.4.1. Geo-Accumulation Index

(1) $$I_{geo}={log}_{2}(\frac{Ci}{1.5\times Bi})$$

where *Ci* is the measured concentration of potentially toxic element *i* in sediment (mg·kg^−1^). *Bi* is the geochemical background reference value for potentially toxic element *i* (mg·kg^−1^), derived from regional pristine sediments (Table S2). The logarithmic multiplier (1.5) serves to normalize inherent lithospheric heterogeneity across geographical regions, thereby enhancing the index’s capacity to differentiate anthropogenic contamination from natural geochemical baselines. The computed $I_{geo}$ values were classified into seven contamination tiers (Table S3), ranging from Class 0 ($I_{geo}$ < 0, uncontaminated) to Class 6 ($I_{geo}$ ≥ 5, extremely contaminated).

#### 2.4.2. Potential Ecological Risk Index 

The potential ecological risk factor of the single-element ecological risk coefficient ($E_{r}^{i}$) was defined as follows:

(2) $$E_{r}^{i}=T_{r}^{i}\times \frac{Ci}{Bi}$$

(3) $$PERI=\sum E_{r}^{i}$$

Similarly, *Ci* is the measured concentration of potentially toxic element *i* in sediment (mg·kg^−1^) and *Bi* is the geochemical background reference value for potentially toxic element *i* (mg·kg^−1^). $T_{r}^{i}$ corresponds to the toxic response factor for potentially toxic element *i*, with values assigned as follows: Pb = 5, Cd = 30, Zn = 1, Cr = 2, Cu = 5, As = 10, Ni = 5, Mn = 1, and Hg = 40 (Table S2). The computed risk indices are classified into five hierarchical tiers according to the method of Hakanson [10] (Table S4).

### 2.5. Statistical Analysis

The effects of aquaculture seasons and patterns on the physicochemical and nutrient characteristics and potentially toxic element composition of sediments were, respectively, assessed using PERMANOVA. Based on NMDS and ADONIS, the influence of aquaculture patterns on sediments’ potentially toxic element composition under the same aquaculture seasons and the influence of aquaculture seasons on sediments’ potentially toxic element composition under the same aquaculture patterns were evaluated. One-way ANOVA was used to compare the significance of differences between different treatment groups. Pearson correlation analysis was employed to explore potential relationships among potentially toxic elements, between potentially toxic elements and environmental factors, and among environmental factors themselves, with results visualized using Gephi (v 0.9.2).

## 3. Results

### 3.1. Physicochemical and Nutrient Characteristics of Sediments

**Figure 1:**
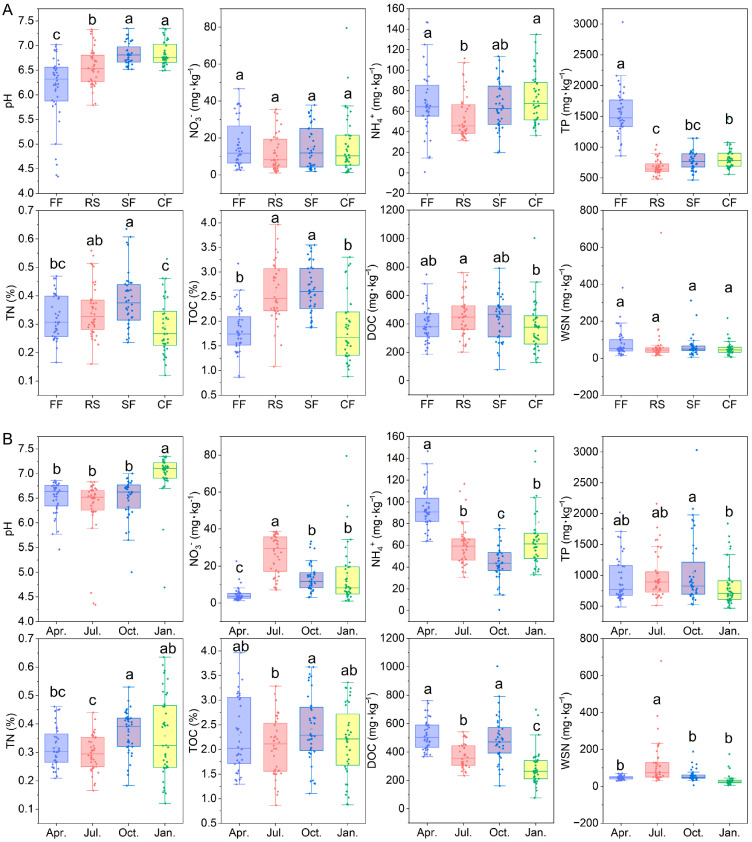
The influence of aquaculture patterns (**A**) and aquaculture seasons (**B**) on the physicochemical and nutrient characteristics of aquaculture pond sediments in Jianghan Plain. Different lowercase letters indicate significant differences (*p*-value < 0.05).

Throughout the entire aquaculture process, the physicochemical and nutrient characteristics of sediments varied among different farming patterns (Figure 1A). The sediment pH in crayfish and crab ponds was significantly higher than that in fish ponds and rice–crayfish co-culture ponds. Fish ponds exhibited the lowest pH values, followed by rice–crayfish co-culture ponds. However, the ammonium nitrogen (NH_4_^+^-N) and total phosphorus (TP) contents in rice–crayfish co-culture ponds were significantly lower than those in fish ponds and crab ponds. The total nitrogen (TN) and total organic carbon (TOC) in crab ponds were significantly lower than those in rice–crayfish co-culture ponds and crayfish ponds, whereas dissolved organic carbon (DOC) in crab ponds was only significantly lower than in rice–crayfish co-culture ponds. Additionally, no significant differences were detected in nitrate nitrogen (NO_3_^−^-N) and water-soluble nitrogen (WSN) among the different aquaculture patterns.

The physicochemical and nutrient characteristics of sediments in all aquaculture ponds also varied across different aquaculture seasons (Figure 1B). The sediment pH in winter (January) was significantly higher than in the other three seasons, while NO_3_^−^-N and WSN contents in July were significantly higher than those in other seasons (Table S5). In April, NO_3_^−^-N content was significantly lower than in other seasons, whereas NH_4_^+^-N was significantly higher than in other seasons. TP in October was only significantly higher than in January, while sediment TN in October was significantly higher than in April and July. TOC in October was significantly higher than in July, and DOC in April and October was significantly higher than in July and January. These results indicate that the physicochemical and nutrient characteristics of aquaculture pond sediments are influenced by both farming patterns and seasonal variations. The contributions of aquaculture patterns and aquaculture seasons to the physicochemical and nutrient characteristics of sediments were further explored (Table 1). It was found that both aquaculture seasons and aquaculture patterns had significant effects on the variation in sediment physicochemical and nutrient characteristics (*p*-value < 0.001), and the contribution of aquaculture patterns (R^2^ = 0.6239) was much greater than that of aquaculture seasons (R^2^ = 0.0885).

### 3.2. Characteristics of Potentially Toxic Element Occurrence in Sediments

**Figure 2:**
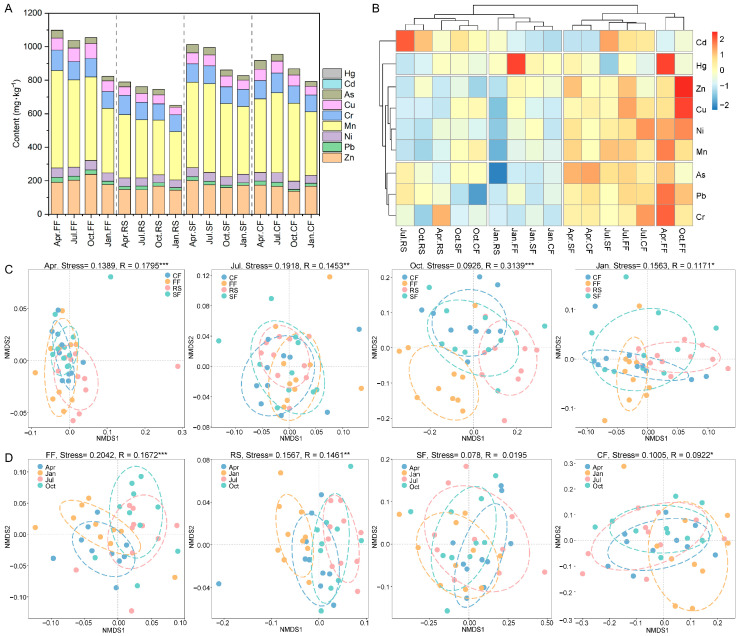
Analysis of potentially toxic element composition and differences in sediment of aquaculture ponds in Jianghan Plain. (**A**) Composition of potentially toxic elements in sediment of aquaculture ponds. (**B**) Cluster heatmap of potentially toxic elements in sediment of aquaculture ponds. (**C**) Effect of aquaculture season on the composition of potentially toxic elements. (**D**) Effect of aquaculture patterns on the composition of potentially toxic elements. * indicates *p*-value < 0.05, ** indicates *p*-value < 0.01, and *** indicates *p*-value < 0.001.

All nine potentially toxic elements (Zn, Pb, Ni, Mn, Cr, Cu, As, Cd, and Hg) were detected in the sediments of all aquaculture ponds (Figure 2A). Mn (mean = 435.42 mg/kg) was the potentially toxic element with the highest concentration across all aquaculture ponds, followed by Zn (mean = 172.69 mg/kg), Cr (mean = 106.79 mg/kg), and Cu (mean = 63.44 mg/kg). Overall, the potentially toxic element content in the sediments of rice–crayfish co-culture ponds was lower than that in the other three aquaculture patterns (Figure 2A,B). In spring (April), the sediment concentrations of Zn and As in rice–crayfish co-culture ponds were significantly lower than those in the other three aquaculture patterns. In summer (July), the sediment concentrations of Mn and Cu in rice–crayfish co-culture ponds were significantly lower than those in the other three aquaculture patterns. In autumn (October), the sediment concentration of Mn in rice–crayfish co-culture ponds was significantly lower than that in the other three aquaculture patterns (Table S6). In winter (January), the sediment concentrations of Mn and As in rice–crayfish co-culture ponds were significantly lower than those in the other three aquaculture patterns. This indicates that the potentially toxic element content in the sediments of aquaculture ponds in the Jianghan Plain is influenced by both aquaculture patterns and aquaculture seasons.

NMDS and ADONIS were used to investigate the influence of different culture patterns on sediments’ potentially toxic element composition within the same seasons, and the influence of different aquaculture seasons on sediments’ potentially toxic element composition under the same aquaculture patterns (Figure 2C,D). Across all four seasons, sediment composition was significantly affected by culture mode (Figure 2C). This effect was strongest in autumn (R = 0.3139) and weakest in spring (R = 0.1171), suggesting that the potentially toxic element composition in sediments of different culture patterns is influenced by seasonal variation. Aquaculture season had the greatest impact on the potentially toxic element composition in fish pond sediments (R = 0.1672), followed by rice–crayfish ponds (R = 0.1461) and crab ponds (R = 0.0922, Figure 2D). Aquaculture season did not have a significant effect on the potentially toxic element composition in crayfish ponds (Figure 2D). This demonstrates that seasonal changes differentially affect the potentially toxic element composition in sediments of different aquaculture patterns.

Furthermore, PERMANOVA analysis was conducted to investigate the contributions of aquaculture patterns and aquaculture seasons to sediments’ potentially toxic element composition (Table 2). It was also found that both aquaculture seasons and aquaculture patterns had significant effects on the variation in sediments’ potentially toxic element composition (*p*-value < 0.001), with the contribution of aquaculture mode (R^2^ = 0.1998) far exceeding that of aquaculture seasons (R^2^ = 0.1099).

### 3.3. Influence of Aquaculture Patterns and Seasons on Potentially Toxic Element Occurrence in Sediments

Throughout the culture cycle, rice–crayfish eco-culture significantly reduced sediment concentrations of Zn, Ni, Mn, Cu, and As. However, Cd levels in rice–crayfish ponds were significantly higher than those in fish ponds and crab ponds (Figure S1). Conversely, fish ponds exhibited significantly elevated concentrations of Zn, Pb, Cu, and Hg compared to the other three aquaculture systems, demonstrating stronger sediment enrichment characteristics.

Significant differences were detected for eight potentially toxic elements (Pb, Ni, Mn, Cr, Cu, As, Cd, and Hg) across aquaculture seasons in pond sediments, while Zn showed no significant variation (Figure S2). During the winter fallow period, significantly lower concentrations of Pb, Ni, Mn, Cu, and As were observed compared to the other three seasons. Autumn/winter ponds had reduced Cr levels relative to spring/summer ponds. Cd exhibited an initial increase followed by a decrease during the culture cycle, returning to spring stocking-period levels by winter fallow. Hg displayed an initial decrease followed by an increase. The Cd trajectory suggests that scientifically planned culture/fallow cycles could leverage the sediment’s natural purification capacity to mitigate Cd risks. This provides a temporal management basis for sustainable aquaculture.

### 3.4. Co-Occurrence Patterns of Potentially Toxic Elements in Sediments and Their Influencing Factors

**Figure 3:**
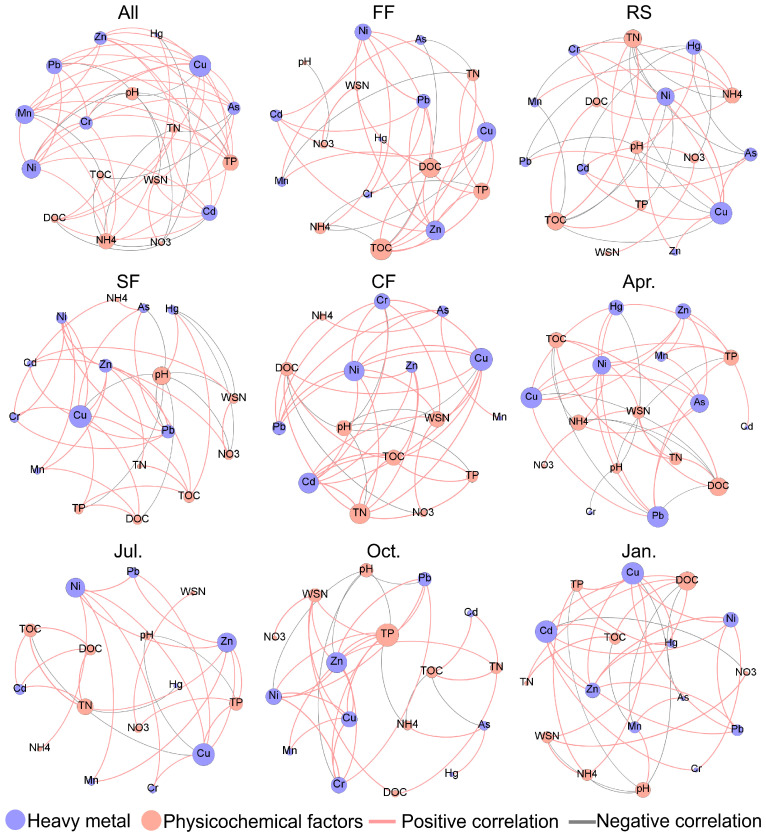
Co-occurrence patterns of potentially toxic elements in aquaculture pond sediments and their influencing factors. The data in the Figure show a significant correlation (*p*-value < 0.05).

There was a significant positive correlation between Zn and Pb, Ni, Mn, Cu, Cd, and Hg in all the fish ponds. Pb has a significant positive correlation with Ni, Mn, Cr, Cu, and As (Figure 3). These results indicate that the potentially toxic elements in the fish ponds occur in multiple co-occurrence patterns. In addition, As has a significant positive correlation with Pb, Ni, Mn, Cu, and Cd. Cd has a significant positive correlation with Ni and Cu (Figure 3). This may suggest that the potentially toxic elements in the pond sediments exhibit distinct geochemical behaviors.

The different potentially toxic elements in the sediment of all the fish ponds are affected by their different physical and chemical properties. The content of Zn in the sediment is mainly affected by TP (*p*-value = 0.358) and TN (*p*-value = 0.202). However, Cd is mainly affected by NH_4_^+^-N, TN, TOC, DOC, and WSN. The content of Cd decreases with the increase in NH_4_^+^-N. In addition, the pH of the sediment has a significant negative correlation with the contents of Pb, Ni, Cr, and Cu. TP has a significant positive correlation with the contents of more potentially toxic elements in the sediment (Zn, Pb, Ni, Mn, Cr, Cu, and Hg), which means that TP may be an important indicator affecting the potentially toxic elements in the aquatic sediment of fish ponds (Figure 3).

However, the co-occurrence patterns of potentially toxic elements and their influencing factors vary in different fish pond management systems. For example, in the fish pond sediment, there is a significant positive correlation between Zn and Pb, Ni, Cu, and Cd, while in the rice–crayfish pond, Zn only has a significant positive correlation with Ni and Cu (Figure 3). In contrast, in the pure crayfish pond, Zn has a significant positive correlation with Pb, Ni, Mn, and Cu (Figure 3). This may suggest that rice–crayfish farming may reduce the co-occurrence pattern of potentially toxic elements in the sediment. The content of Zn, Ni, Mn, and Cu in the fish pond sediment has a significant positive correlation with dissolved organic carbon, while DOC in the rice–crayfish pond only has a significant positive correlation with Cr, and DOC in the crab pond only has a significant positive correlation with Cu (Figure 3).

Furthermore, the co-occurrence patterns of potentially toxic elements and their influencing factors are also affected by the farming season. During the spring stocking period, Cd has a significant positive correlation with Zn, while during the summer and autumn farming periods, Cd does not show a significant positive correlation with other potentially toxic elements. During the winter fishing period, Cd has a significant positive correlation with Zn, Pb, Ni, and Cr (Figure 3). This indicates that the co-occurrence patterns of potentially toxic elements in the fish ponds vary at different farming stages. TP is an important factor affecting the potentially toxic elements in the fish ponds in spring and autumn, while potentially toxic elements are mainly affected by TN, TP, and TOC in summer, and by DOC in winter.

### 3.5. Potential Ecological Risk Assessment of Potentially Toxic Elements in Sediments

The geo-accumulation index (I_geo_) indicates that Pb and Mn in all aquaculture ponds were in an uncontaminated state (I_geo_ ≤ 0) (Table 3). Cr, Hg, and Ni were in an uncontaminated state (I_geo_ ≤ 0) in 98.125%, 97.5%, and 77.5% of aquaculture ponds, respectively. Conversely, Cr and Hg were in an uncontaminated to moderately contaminated state (0 < I_geo_ ≤ 1) in 1.875%, 2.5%, and 22.5% of aquaculture ponds, respectively. In comparison, Cd, Cu, As, and Zn were in an uncontaminated to moderately contaminated state (0 < I_geo_ ≤ 1) in 97.5%, 89.375%, 88.125%, and 78.75% of aquaculture ponds, respectively.

**Figure 4:**
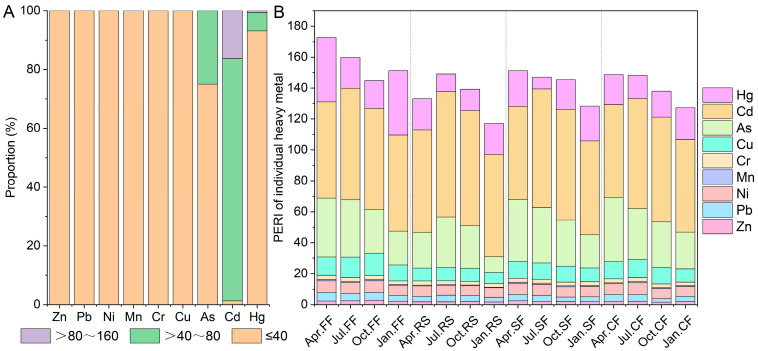
Potential ecological risk index of individual elements ($E_{r}^{i}$). (**A**) Proportions of aquaculture ponds with low risk ($E_{r}^{i}$ ≤ 40), moderate risk (40 < $E_{r}^{i}$ ≤ 80), and considerable risk (80 < $E_{r}^{i}$ ≤ 160) for different potentially toxic elements. (**B**) Potential ecological risk indices of various elements in sediment for different aquaculture modes and different aquaculture seasons.

The single-element potential ecological risk index indicates ($E_{r}^{i}$) that all aquaculture ponds exhibited low risk ($E_{r}^{i}$ ≤ 40) from Zn, Pb, Ni, Mn, Cr, and Cu (Figure 4A). A total of 25.00% of sediment samples showed moderate risk ($E_{r}^{i}$ > 40 to $E_{r}^{i}$ ≤ 80) from As. A total of 82.50% and 16.25% of sediment samples demonstrated moderate risk and considerable risk ($E_{r}^{i}$ > 80 to $E_{r}^{i}$ ≤ 160) from Cd, respectively. Similarly, 6.25% and 0.63% of sediment samples exhibited moderate risk and considerable risk from Hg, respectively. The potential ecological risks of potentially toxic elements in aquaculture pond sediments were mainly contributed by metals such as Cd, As, Hg, and Cu (Figure 4B).

**Figure 5:**
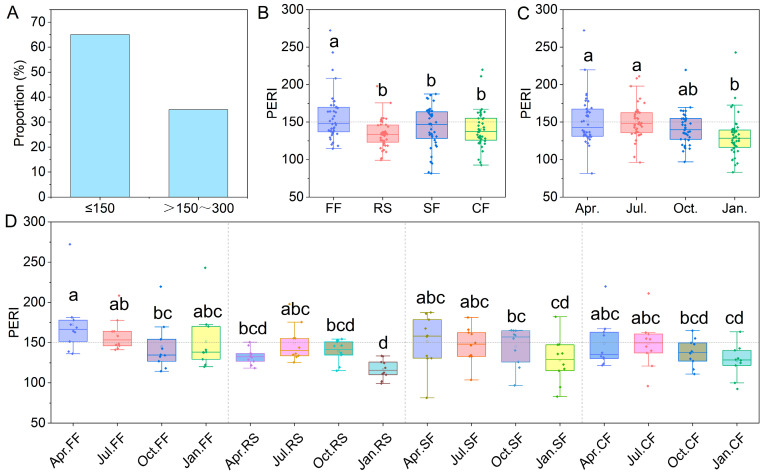
Integrated potential ecological risk index (PERI). (**A**) Proportion of aquaculture ponds with low-risk (PERI < 150) and moderate-risk (150 ≤ PERI < 300) PERI. (**B**) PERI contributions from different aquaculture patterns. (**C**) PERI contributions from different aquaculture seasons. (**D**) PERI contributions from the interaction of aquaculture patterns and aquaculture seasons. Different lowercase letters indicate significant differences (*p*-value < 0.05).

The integrated potential ecological risk index (PERI) revealed that 65.00% of aquaculture ponds exhibited low ecological risk (PERI < 150), while 35% showed moderate ecological risk (150 ≤ PERI < 300) in their sediments (Figure 5A). Overall, fish ponds demonstrated significantly higher PERI values than the other three aquaculture systems (Figure 5B). Furthermore, sediment PERI decreased progressively during the culture cycle (Figure 5C), with this trend being particularly pronounced in crayfish and crab ponds (Figure 5D). However, rice–crayfish co-culture ponds displayed a distinct PERI trajectory: initially increasing, then decreasing over the culture period (Figure 5D). Crucially, during equivalent culture stages, these ponds consistently maintained lower PERI values than the other three systems, with differences becoming most evident during the January fallow period.

## 4. Discussion

Understanding the occurrence characteristics of potentially toxic elements in aquaculture pond sediments and their potential ecological risks is essential for assessing environmental pollution, ensuring the safety of aquatic products, and guiding pollution control [4]. This will contribute to protecting the ecological environment and achieving sustainable aquaculture development. However, current research on potentially toxic element pollution in freshwater aquaculture ponds mostly focuses on single sampling events in individual aquaculture patterns, lacking comprehensive comparative analyses across different aquaculture patterns and long-term monitoring. Therefore, this study systematically investigates the different aquaculture season occurrence characteristics, potential ecological risks, and driving factors of potentially toxic elements in sediments under four distinct aquaculture patterns (fish ponds, rice–crayfish co-culture ponds, crayfish ponds, and crab ponds). It reveals the multifaceted impacts of farming activities on the aquatic environment and provides critical science-based insights.

### 4.1. Potentially Toxic Element Pollution in the Sediments of Aquaculture Ponds Presents Multiple and Complex Occurrence Patterns

Sediment quality in aquaculture ponds is critical for aquatic product safety and human health [4,5]. The substantial input of uneaten feed and fecal matter resulting from increased stocking density significantly exacerbates sediment pollution risks [4,13]. This study confirmed that nine metals (Zn, Pb, Ni, Mn, Cr, Cu, As, Cd, and Hg) were ubiquitously present in sediments from all investigated aquaculture ponds across the Jianghan Plain. The phenomenon is supported by regional surveys of potentially toxic elements in lake water bodies and sediments [14]. Mn was the most abundant element in pond sediments, which is consistent with observations from coastal aquaculture farms in the northern Bay of Bengal [15].

However, geo-accumulation index (I_geo_) assessments revealed that while Pb (100.00%), Mn (100.00%), Cr (98.13%), Hg (97.50%), and Ni (77.50%) were in an uncontaminated state in the vast majority of aquaculture ponds, over 75.00% of aquaculture ponds exhibited uncontaminated to moderately contaminated levels (0 < I_geo_ ≤ 1) for Cd, Cu, As, and Zn. Potential ecological risk assessment further identified moderate risk in 35% of aquaculture ponds, primarily driven by Cd with significant contributions from Hg, As, and Cu. This pattern aligns with recent regional studies in the Jianghan Plain, which have identified Cd as a pollutant of widespread concern. For instance, research on rice–shrimp co-culture ponds identified Cd, Ni, Zn, and Cu as the primary elements for slight enrichment, with Cd posing a moderate ecological risk at over half of the sites [16]. Extending this finding to a broader aquatic context, a study on lake sediments within the same plain confirmed that Cd contributed the most (43.4%) to the overall potential ecological risk [14]. Collectively, these results demonstrate that potentially toxic element contamination in Jianghan Plain aquaculture pond sediments exhibits complex characteristics: ubiquity, multi-element coexistence, and differentiated pollution levels. The potential ecological risk stems mainly from the combined effects of multiple metals rather than individual contaminants.

Correlation analysis revealed complex co-occurrence patterns among potentially toxic elements. There are significant positive correlations between Zn and Pb, Ni, Mn, Cu, Cd, and Hg in all ponds. There are significant positive correlations between Pb and Ni, Mn, Cr, Cu, and As. There are significant positive correlations among As, Pb, Ni, Mn, Cu, and Cd, and between Cd and Ni and Cu. This result was similar to research on Jianghan Plain wetland sediments, which also confirmed extremely significant positive correlations between Hg, Cr, and Ni, as well as significant positive correlations between Cd, Pb, and Zn [17]. These observed co-occurrence patterns strongly suggest shared pollution sources or similar geochemical behaviors for these metal groups. Integrating these findings with the existing literature, the multi-metal contamination in the ponds can be attributed to several pathways: (1) Aquaculture activities: Feed additives (introducing Cu, Zn, and As) and fertilizer/manure application (introducing Cd, Cu, Zn, and As) [4,5]; (2) peripheral agricultural practices: Pesticide/herbicide use (especially in rice–crayfish ponds, introducing Cu, legacy Hg, and As); (3) geogenic background: Primarily contributing Cr, Ni, and Mn. This multi-source apportionment aligns with conclusions that Cd, Pb, Cr, and Zn in freshwater fish pond sediments are predominantly influenced by anthropogenic sources [4], and that Cr, Cu, and Zn likely originate from anthropogenic inputs [15].

### 4.2. Breeding Pattern Influence Exceeds Seasonal Variations on Potentially Toxic Elements

The Jianghan Plain, a vital agricultural and aquaculture region in China, is highly suitable for diverse aquaculture industries due to its abundant water resources and favorable ecological conditions [18]. Specifically, fish farming, crab farming, crayfish farming, and rice–crayfish co-culture are widely practiced here. This study reveals that the distribution characteristics of potentially toxic elements in aquaculture sediments across the Jianghan Plain are primarily governed by differences in aquaculture practices, with their influence far exceeding that of seasonal variation. Research demonstrates that distinct aquaculture practices (fish, crab, crayfish, and rice–crayfish co-culture) directly shape sediment environments through anthropogenic management practices, including feed formulations containing Zn/Cu additives, pesticide application in rice–crayfish systems, water regulation intensity, and bioturbation. For instance, fish ponds exhibited a significantly higher integrated ecological risk index in sediments compared to other systems, likely due to inputs of high-protein feed. Conversely, rice–crayfish co-culture showed reduced potentially toxic element concentrations and lower risk indices, potentially mediated through processes like rhizosphere effects. PERMANOVA analysis further confirmed that aquaculture practices explained nearly twice the variance in potentially toxic elements (R^2^ = 0.1998) compared to seasonal effects (R^2^ = 0.1099), underscoring the dominant role of management modes.

Seasonal variations in rainfall and human activities reportedly influence potentially toxic element pollution in aquaculture environments [19], while this study found that seasonal changes primarily act as modulators of aquaculture practice effects rather than the dominant driver, though potentially affecting metal mobility via rainfall and temperature fluctuations (e.g., decreased metal levels in winter). The key evidence is reflected in three aspects. First, the differences among the models reached the maximum separation intensity (R = 0.3139) during the peak breeding period in October, while the differences still persisted when the seasonal effect was weakest in January. Second, the crayfish ponds, due to the management of closed water bodies, completely buffered the seasonal impact (with no significant changes in the composition of potentially toxic elements), while the crab ponds and rice–crayfish ponds showed only moderate responses, and only the open fish ponds were significantly affected by the seasons (R = 0.1672). Third, the seasonal impact on potentially toxic elements presented a fragmented trend (such as Cd rising first and then falling, and Hg falling first and then rising), while the breeding models formed stable pollution characteristics through global regulatory mechanisms. For example, rice–crayfish ponds maintained consistently low TP loading, with TP showing significant positive correlations with seven potentially toxic elements. Therefore, optimizing aquaculture practices proves substantially more effective than managing seasonal variations for controlling potentially toxic element risks.

### 4.3. Rice–Crayfish Farming Reduces Sediments’ Potentially Toxic Element Content and Potential Risks

Rice–crayfish co-culture is a prevalent integrated rice-aquaculture system in the middle and lower reaches of the Yangtze River, China. Approximately 140,000 hectares of land in Hubei Province are dedicated to this practice [20]. Studies report that potentially toxic element concentrations in rice paddy aquaculture environments are generally higher than in rice–crayfish co-culture systems, with particularly superior arsenic (As) pollution control in the latter [21]. This study demonstrates that the rice–crayfish co-culture model reduces both potentially toxic element concentrations in sediments and their potential ecological risk. Specifically, beyond mitigating As contamination, this system significantly suppresses pollution from Zn, Ni, Mn, and Cu. This reduction is directly reflected in the progressive optimization of the integrated ecological risk index (PERI): PERI values in rice–crayfish ponds remained the lowest year-round, and these differences were most pronounced during the fallow period (January). Although risk slightly increased during initial culture stages, peak values remained lower than in other systems. This confirms the rice–crayfish co-culture system’s long-term risk regulation capacity. Direct comparative research has further substantiated that the metal pollution index in rice–crayfish co-culture systems is significantly lower than in intensive crayfish monoculture systems, underscoring the inherent environmental advantage of the integrated ecological model over single-species intensive farming [7].

The advantage of rice–crayfish co-culture in reducing potentially toxic element pollution stems from the dual optimization of ecological synergy within the system and human management. On the one hand, the roots of rice secrete organic acids (such as citric acid and oxalic acid), which strongly accumulate and immobilize potentially toxic elements like Cd and As in the sediment [22,23]. On the other hand, this model may significantly reduce pollution input through control of three major sources. (a) In the rice–crayfish farming model, only feeding is required during the crayfish-farming period, thereby reducing the introduction of potentially toxic elements (containing Cu/Zn) in the feed additives [7]; (b) During the rice cultivation period, chemical pesticides are prohibited to prevent the death of the crayfish, thereby avoiding potentially toxic element pollutants such as As; (c) The phosphorus excreted by the crayfish is absorbed by the rice, resulting in a lower TP load compared to fish ponds [24] and reducing the potentially toxic element co-precipitation mediated by TP [25]. Especially in rice–crayfish co-culture, the accumulation of toxic elements in crayfish is less [26]. Thus, this “combining biological uptake, pollution interception, and nutrient cycling” three-in-one natural solution provides a sustainable path for the prevention and control of potentially toxic elements in aquaculture.

### 4.4. TP Serves as a Critical Regulator of Potentially Toxic Element Occurrence in Aquaculture Pond Sediments

This study revealed significant positive correlations between TP and seven potentially toxic elements (Zn, Pb, Ni, Mn, Cr, Cu, and Hg), suggesting that phosphorus loading may exert broad-spectrum regulatory effects on metal accumulation.

This strong correlation may stem from three synergistic drivers. Firstly, the high TP environment promotes the co-precipitation of metals through phosphate adsorption or the formation of stable compounds (such as Zn_3_(PO_4_)_2_) [25]; secondly, TP is often coupled with total organic matter (TOC/DOC), and the organic-phosphorus complexes formed can effectively fix metal ions [27]; thirdly, the TP level directly reflects the intensity of human activities, such as excessive feeding and fecal discharge [24]. Feed residues and fecal waste containing crude protein and trace metal additives (e.g., Cu, Zn) elevate both TP and potentially toxic element loads concurrently [19], establishing TP as an integrated indicator of potentially toxic element pollution input.

The rice–crayfish co-culture system demonstrates the importance of phosphorus management in mitigating potentially toxic element risks through source reduction in TP loading. Compared to other aquaculture ponds (e.g., fish ponds, crab ponds), rice–crayfish ponds exhibit significantly lower TP concentrations. This reduced TP environment diminishes metal activation and retention capacity, resulting in a significantly lower integrated ecological risk index (PERI) than other systems. Additional advantages include reduced feed inputs and enhanced phosphorus recycling [7,24]. Therefore, controlling phosphorus loading may constitute a key strategy for reducing potentially toxic element accumulation in aquaculture pond sediments and associated ecological risks via measures such as optimized feed management and enhanced sediment dredging.

## 5. Conclusions

This study reveals the distribution characteristics of potentially toxic elements in sediments of aquaculture ponds under the dynamic interaction of aquaculture patterns and seasonal changes, with culture type being the dominant factor: fish farming promotes heavy metal accumulation, whereas rice–crayfish co-culture effectively reduces such enrichment and ecological risks. TP, as a key driver, significantly correlated with multiple potentially toxic elements, indicating that its management is crucial for pollution control. The pollution profile exhibits a “multi-contaminant state” dominated by Cd, Cu, As, Zn, and Hg, necessitating integrated control strategies targeting these priority metals and TP. Several research gaps remain, including the bioavailability of potentially toxic elements within the system, their food-chain transfer mechanisms, and the long-term coupling processes between TP and potentially toxic elements. Future studies should focus on multi-media (sediment–water–biota) approaches to clarify potentially toxic element speciation, transport, and transformation mechanisms. Through long-term monitoring and the development of remediation technologies, a precision-based prevention and control framework suitable for aquaculture systems should be established, providing theoretical and technical support for the achievement of green and sustainable aquaculture.

## Figures and Tables

**Table 1:** The contribution of aquaculture patterns and aquaculture seasons to the physicochemical and nutrient characteristics of sediments.

	R^2^	*p*-Value
Aquaculture seasons	0.0885	0.0001
Aquaculture patterns	0.6239	0.0001
Aquaculture seasons × Aquaculture patterns	0.0153	0.5572
Residuals	0.2723	

**Table 2:** The contribution of aquaculture patterns and aquaculture seasons to the potentially toxic element composition of sediments.

	R^2^	*p*-Value
Aquaculture seasons	0.1099	0.0001
Aquaculture patterns	0.1998	0.0001
Aquaculture seasons × Aquaculture patterns	0.0404	0.4572
Residuals	0.6499	

**Table 3:** The proportion of aquaculture ponds at different pollution levels in the Jianghan Plain based on the geo-accumulation index (I_geo_).

Pollution Levels	Values	Pollution Degree	The Proportion of Aquaculture Ponds at Different Pollution Levels (%)								
			Zn	Pb	Ni	Mn	Cr	Cu	As	Cd	Hg
Class 0	$I_{geo}$ ≤ 0	Uncontaminated	21.250	100.000	77.500	100.000	98.125	10.625	11.875	2.500	97.500
Class 1	$0\leq I_{geo}$ < 1	Uncontaminated to moderately contaminated	78.750	0.000	22.500	0.000	1.875	89.375	88.125	97.500	2.500
Class 2	$1\leq I_{geo}$ < 2	Moderately contaminated	—	—	—	—	—	—	—	—	—
Class 3	$2\leq I_{geo}$ < 3	Moderately to heavily contaminated	—	—	—	—	—	—	—	—	—
Class 4	$3\leq I_{geo}$ < 4	Heavily contaminated	—	—	—	—	—	—	—	—	—
Class 5	$4\leq I_{geo}$ < 5	Heavily to extremely contaminated	—	—	—	—	—	—	—	—	—
Class 6	$I_{geo}$ ≥ 5	Extremely contaminated	—	—	—	—	—	—	—	—	—

## Data Availability

The raw data supporting the conclusions of this article will be made available by the authors on request.

## Supplementary Materials

The following supporting information can be downloaded at https://www.mdpi.com/article/10.3390/toxics14010065/s1: Figure S1: Effects of different farming patterns on the contents of nine potentially toxic elements in aquaculture pond sediments. Different lowercase letters indicate significant differences at the 0.05 level; Figure S2: Effects of different aquaculture seasons on the contents of nine potentially toxic elements in sediment samples from aquaculture ponds. Different lowercase letters indicate significant differences at the 0.05 level; Table S1: Geographical information of sampling sites; Table S2: Toxicity response coefficients of various potentially toxic elements and reference standards for potentially toxic elements (mg/kg); Table S3: Criteria for geoaccumulation index (*Igeo*); Table S4: Critical range and grades of $E_{r}^{i}$ and PERI; Table S5: Physicochemical and nutritional characteristics of sediments in aquaculture ponds of the Jianghan Plain. Different lowercase letters indicate significant differences (*p*-value < 0.05) among different aquaculture patterns within the same aquaculture season. Different uppercase letters indicate significant differences (*p*-value < 0.05) among different aquaculture season within the same culture aquaculture patterns; Table S6: Potentially toxic element content in sediments of aquaculture ponds in the Jianghan Plain. Different lowercase letters indicate significant differences (*p*-value < 0.05) among different aquaculture patterns within the same aquaculture season. Different uppercase letters indicate significant differences (*p*-value < 0.05) among different aquaculture season within the same aquaculture patterns.

## References

[1] Emenike E.C., Iwuozor K.O., Anidiobi S.U. (2022). Heavy metal pollution in aquaculture: Sources, impacts and mitigation techniques. Biol. Trace Elem. Res..

[2] Peng W., Li X., Xiao S., Fan W. (2018). Review of remediation technologies for sediments contaminated by heavy metals. J. Soils Sediments.

[3] Shi J., Wu X., Zhao X., Zhou J., Liu S., Li B., Zhang J., Li W., Zeng X., Wang X. (2024). Remediation of heavy metal-contaminated estuarine sediments by strengthening microbial in-situ mineralization. Appl. Geochem..

[4] Liu S., Wu K., Yao L., Li Y., Chen R., Zhang L., Wu Z., Zhou Q. (2024). Characteristics and correlation analysis of heavy metal distribution in China’s freshwater aquaculture pond sediments. Sci. Total Environ..

[5] Kong W., Xu Q., Lyu H., Kong J., Wang X., Shen B., Bi Y. (2023). Sediment and residual feed from aquaculture water bodies threaten aquatic environmental ecosystem: Interactions among algae, heavy metals, and nutrients. J. Environ. Manag..

[6] Yuan G.L., Liu C., Chen L., Yang Z. (2011). Inputting history of heavy metals into the inland lake recorded in sediment profiles: Poyang Lake in China. J. Hazard. Mater..

[7] Mo A., Dang Y., Wang J., Liu C., Yang H., Zhai Y., Wang Y., Yuan Y. (2022). Heavy metal residues, releases and food health risks between the two main crayfish culturing models: Rice-crayfish coculture system versus crayfish intensive culture system. Environ. Pollut..

[8] Yadav N.K., Patel A.B., Singh S.K., Mehta N.K., Anand V., Lal J., Dekari D., Devi N.C. (2024). Climate change effects on aquaculture production and its sustainable management through climate-resilient adaptation strategies: A review. Environ. Sci. Pollut. Res. Int..

[9] Alvarez M.B., Quintas P.Y., Domini C.E., Garrido M., Fernandez B. (2014). Chemometric approach to visualize and easily interpret data from sequential extraction procedures applied to sediment samples. J. Hazard. Mater..

[10] Hakanson L. (1980). An ecological risk index for aquatic pollution-control—A sedimentological approach. Water Res..

[11] APHA (2017). Standard Methods for the Examination of Water and Wastewater.

[12] Rodrigues P.A., Almeida A.C.O., Ramos-Filho A.M., Braz B.F., Santelli R.E., Conte-Junior C.A. (2025). Assessment of toxic and potentially toxic elements in fishery species in the southeastern region of Brazil: Environmental correlations, biometrics, and human health risk. Mar. Pollut. Bull..

[13] Shen X., Xu M., Li M., Zhao Y., Shao X. (2020). Response of sediment bacterial communities to the drainage of wastewater from aquaculture ponds in different seasons. Sci. Total Environ..

[14] Wang C., Wang K., Zhou W., Li Y., Zou G., Wang Z. (2023). Occurrence, risk, and source of heavy metals in lake water columns and sediment cores in Jianghan Plain, Central China. Int. J. Environ. Res. Public Health.

[15] Hossain M.B., Sultana J., Pingki F.H., Nur A.A.U., Mia M.S., Abu Bakar M., Yu J., Paray B.A., Arai T. (2023). Accumulation and contamination assessment of heavy metals in sediments of commercial aquaculture farms from a coastal area along the northern Bay of Bengal. Front. Environ. Sci..

[16] Liu B., Xiong J., Guo L., Yu K., Yao S. (2023). Heavy metals and as enrichment of sediments in typical shrimp-rice co-cropping culture ponds at Jianghan Plain. Fish. Sci..

[17] Wang W., Gu D.S., Ding J.J. (2023). Risk assessment of heavy metal pollution from wetland sediments in Xiantao Area, Jianghan Plain. Saf. Environ. Eng..

[18] Wang C., Dong J., Zhou Y., Cui Y., Chen X., Di Y., Xiao X., Zhang G. (2025). Satellite observed rapid inland aquaculture expansion in Jianghan Plain, China from 2016 to 2022. Aquaculture.

[19] Li J., Cui H., Guo Y., Li P., Han J., Li W. (2023). Aquaculture exacerbates the accumulation and ecological risk of heavy metal from anthropogenic and natural sources, a case study in Hung-tse Lake, China. Water Air Soil Pollut..

[20] Si G., Peng C., Yuan J., Xu X., Zhao S., Xu D., Wu J. (2017). Changes in soil microbial community composition and organic carbon fractions in an integrated rice-crayfish farming system in subtropical China. Sci. Rep..

[21] Peng F., Yang F., Du H. (2024). Comparisons of heavy metals levels in water, sediment and crayfish under rice-crayfish co-culture and pond culture modes with correlation analysis and health risk assessment. J. Food Meas. Charact..

[22] Islam M.S., Kormoker T., Idris A.M., Proshad R., Kabir M.H., Ustaoğlu F. (2022). Plant–microbe–metal interactions for heavy metal bioremediation: A review. Crop Pasture Sci..

[23] Zeng F., Chen S., Miao Y., Wu F., Zhang G. (2008). Changes of organic acid exudation and rhizosphere pH in rice plants under chromium stress. Environ. Pollut..

[24] Li Q., Xu L., Xu L., Qian Y., Jiao Y., Bi Y., Zhang T., Zhang W., Liu Y. (2018). Influence of consecutive integrated rice–crayfish culture on phosphorus fertility of paddy soils. Land Degrad. Dev..

[25] Wang Y., Wang X., Li J., Li Y., Liu Y., Wang F., Zhao J. (2020). Adsorption and precipitation behaviors of zinc, copper and tetracycline with struvite products obtained by phosphorus recovery from swine wastewater. J. Environ. Chem. Eng..

[26] Tan Y., Peng B., Wu Y., Xiong L., Sun J., Peng G., Bai X. (2021). Human health risk assessment of toxic heavy metal and metalloid intake via consumption of red swamp crayfish (*Procambarus clarkii*) from rice-crayfish co-culture fields in China. Food Control.

[27] Liu Y.P., Song D., Jiao L.X., Zheng J.L., Zhang M., Yao B., Yan J.Y., Wu J.X., Wen X. (2025). Sedimentary differentiation characteristics of organic matter and phosphorus in eutrophic lake special zones. Water.

