# Supplementary material for: Dominant Role of Aquaculture Patterns over Seasonal Variations in Controlling Potentially Toxic Elements’ Occurrence and Ecological Risks in Sediments
Source: Toxics. 2026 Jan 10;14(1):65. doi: 10.3390/toxics14010065 (PMC12846250; doi:10.3390/toxics14010065)
Supplement: Supplementary file 1 [file toxics-14-00065-s001.zip › toxics-4056772-supplementary.pdf]

Supplementary materials

Dominant Role of Aquaculture Patterns over Seasonal Variations in Controlling Potentially Toxic Elements' Occurrence and Ecological Risks in Sediments

Luna Zhang[1,2,3,4;] Yuyi Yang[5,6;] Huabao Zheng[1,*;] Zhi Wang[7;] Weihong Zhang[2,3,4,*]

[1] Key Laboratory of Soil Contamination Bioremediation of Zhejiang Province, College of Environmental and Resource Sciences, Zhejiang Agriculture and Forestry University, Hangzhou 311300, China

[2] State Key Laboratory of Lake and Watershed Science for Water Security, Wuhan Botanical Garden, Chinese Academy of Sciences, Wuhan 430074, China

[3] Hubei Key Laboratory of Wetland Evolution & Ecological Restoration, Wuhan Botanical Garden, Chinese Academy of Sciences, Wuhan 430074, China

[4] Danjiangkou Wetland Ecosystem Field Scientific Observation and Research Station, the Chinese Academy of Sciences & Hubei Province, Wuhan 430074, China

[5] Hubei Key Laboratory of Microbial Transformation and Regulation of Biogenic Elements in the Middle Reaches of the Yangtze River, School of Environmental Ecology and Biological Engineering, Wuhan Institute of Technology, Wuhan 430205, China

[6] State Key Laboratory of Green and Efficient Development of Phosphorus Resources, Wuhan Institute of Technology, 206 Guanggu 1st road, Wuhan 430205, China

[7] Key Laboratory for Environment and Disaster Monitoring and Evaluation of Hubei, Innovation Academy for Precision Measurement Science and Technology, Chinese Academy of Sciences, Wuhan, 430077, China

Corresponding Author: zhenghuabao@zafu.edu.cn (H.Z.); zhangweihong@wbgcas.cn (W.Z.);

Tel.: +86-13186962261 (H.Z.); +86-27-87700853 (W.Z.); Fax: +86-27-87700877 (W.Z.)

Table S1. Geographical information of sampling sites

Samples	Latitude	Longitude	Samples	Latitude	Longitude
FF01	30.06328687°	113.7336547°	RS01	29.91593327°	113.49891359°
FF02	30.06355999°	113.73379677°	RS02	29.90699198°	113.49151093°
FF03	30.06377497°	113.73377578°	RS03	29.92139269°	113.03096442°
FF04	30.07129168°	113.73878464°	RS04	29.92103786°	113.03102861°
FF05	30.07172044°	113.73559303°	RS05	29.92034189°	113.03068436°
FF06	30.05727997°	113.6702219°	RS06	29.91581243°	113.02913053°
FF07	30.05611741°	113.67414031°	RS07	29.91578438°	113.02911146°
FF08	30.05532286°	113.67542839°	RS08	29.91593663°	113.02707527°
FF09	30.06346859°	113.73583437°	RS09	29.91593362°	113.02707025°
FF10	30.06347089°	113.73491912°	RS10	29.91593772°	113.49609001°
SF01	29.9159389°	113.4973703°	CF01	29.91547759°	113.50279013°
SF02	29.91578022°	113.49892951°	CF02	29.9156473°	113.5035513°
SF03	29.91585988°	113.4980438°	CF03	29.91565546°	113.50416302°
SF04	29.91566581°	113.50072783°	CF04	29.91588055°	113.49948546°
SF05	29.91583539°	113.50816108°	CF05	29.91553262°	113.50410622°
SF06	29.91686788°	113.50800675°	CF06	29.91500955°	113.51744245°
SF07	29.91521647°	113.51211127°	CF07	29.91513986°	113.51801335°
SF08	29.91525975°	113.51235137°	CF08	29.9151748°	113.51534224°
SF09	29.91541861°	113.51106818°	CF09	29.90829684°	113.49730984°
SF10	29.90857017°	113.49953762°	CF10	29.90821664°	113.49904342°

Table S2. Toxicity response coefficients of various potentially toxic elements and reference standards for potentially toxic elements (mg/kg).

	Zn	Pb	Ni	Mn	Cr	Cu	As	Cd	Hg
T_r^i	1	5	5	1	2	5	10	30	40
Background	83.60	26.70	37.30	712.00	86.60	30.70	12.30	0.17	0.08

Table S3. Criteria for geoaccumulation index (I_{geo})

Pollution levels	Values	Pollution degree
Class 0	$I_{geo} \leq 0$	Uncontaminated
Class 1	$0 \leq I_{geo} < 1$	Uncontaminated to moderately contaminated
Class 2	$1 \leq I_{geo} < 2$	Moderately contaminated
Class 3	$2 \leq I_{geo} < 3$	Moderately to heavily contaminated
Class 4	$3 \leq I_{geo} < 4$	Heavily contaminated
Class 5	$4 \leq I_{geo} < 5$	Heavily to extremely contaminated
Class 6	$I_{geo} \geq 5$	Extremely contaminated

Table S4. Critical range and grades of E_r^i and *PERI* (Hakanson, 1980)

E_r^i	*PERI*	Risk degree
$E_r^i \leq 40$	PERI<150	Low
$40 < E_r^i \leq 80$	150≤PERI<300	Moderate
$80 < E_r^i \leq 160$	300≤PERI<600	Considerable
$160 < E_r^i \leq 320$	600≤PERI<1200	High
$E_r^i > 320$	PERI ≥1200	Very high

Table S5. Physicochemical and nutritional characteristics of sediments in aquaculture ponds of the Jianghan Plain. Different lowercase letters indicate significant differences (p-value < 0.05) among different aquaculture patterns within the same aquaculture season. Different uppercase letters indicate significant differences (p-value < 0.05) among different aquaculture season within the same culture aquaculture patterns.

		FF	RS	SF	CF
Apr.	pH	6.191 ± 0.109 cAB	6.416 ± 0.105 bB	6.716 ± 0.025 aBC	6.752 ± 0.028 aB
	NO_3-N (mg/kg)	4.554 ± 0.606 aC	5.401 ± 1.980 aB	3.583 ± 0.322 aC	5.106 ± 1.309 aC
	NH_4-N (mg/kg)	94.877 ± 8.440 aA	87.159 ± 4.994 aA	91.797 ± 3.160 aA	100.989 ± 5.996 aA
	TP (mg/kg)	1448.850 ± 93.013 aB	687.700 ± 42.838 bA	829.375 ± 61.245 bA	822.225 ± 80.445 bA
	TN (%)	0.282 ± 0.016 bB	0.327 ± 0.020 abA	0.373 ± 0.020 aA	0.290 ± 0.024 bAB
	TOC (mg/kg)	1.792 ± 0.168 bA	2.690 ± 0.211 aA	2.695 ± 0.207 aAB	1.981 ± 0.218 bA
	DOC (mg/kg)	441.812 ± 21.571 cAB	592.228 ± 34.978 aA	554.55 ± 26.101 abA	493.957 ± 30.273 bcA
	WSN (mg/kg)	45.772 ± 2.680 aB	48.327 ± 4.524 aA	52.343 ± 2.056 aB	42.805 ± 2.717 aB
Jul.	pH (mg/kg)	5.670 ± 0.277 bB	6.419 ± 0.052 aB	6.651 ± 0.035 aC	6.645 ± 0.033 aC
	NO_3-N (mg/kg)	29.936 ± 3.707 aA	22.290 ± 3.288 aA	27.927 ± 2.800 aA	26.243 ± 3.245 aA
	NH_4-N (mg/kg)	59.163 ± 4.090 abB	48.158 ± 3.043 bB	64.625 ± 7.771 abB	66.414 ± 6.611 aB
	TP (mg/kg)	1535.425 ± 94.591 aB	716.175 ± 46.182 bA	832.725 ± 31.965 bA	862.175 ± 38.965 bA
	TN (%)	0.264 ± 0.016 bB	0.312 ± 0.016 abA	0.338 ± 0.015 aA	0.277 ± 0.025 bB
	TOC (mg/kg)	1.635 ± 0.143 bA	2.488 ± 0.161 aA	2.351 ± 0.112 aB	1.744 ± 0.189 bA
	DOC (mg/kg)	358.793 ± 33.029 abBC	410.558 ± 26.845 aB	412.615 ± 23.178 aB	318.29 ± 16.464 bB
	WSN (mg/kg)	136.982 ± 33.339 aA	131.322 ± 62.578 aA	120.168 ± 27.187 aA	86.288 ± 16.849 aA
Oct.	pH	6.148 ± 0.165 bAB	6.375 ± 0.098 bB	6.800 ± 0.035 aB	6.741 ± 0.041 aBC
	NO_3-N (mg/kg)	16.811 ± 2.834 aB	10.974 ± 2.662 aB	15.498 ± 2.431 aB	12.045 ± 1.537 aBC
	NH_4-N (mg/kg)	40.380 ± 7.870 aB	43.835 ± 3.742 aB	43.691 ± 5.022 aC	53.067 ± 5.014 aB
	TP (mg/kg)	1871.050 ± 150.05 aA	678.050 ± 39.574 bA	770.825 ± 35.102 bAB	860.100 ± 37.636 bA
	TN (%)	0.353 ± 0.017 aA	0.365 ± 0.018 aA	0.406 ± 0.022 aA	0.362 ± 0.034 aA
	TOC (mg/kg)	2.071 ± 0.120 bA	2.739 ± 0.189 aA	2.848 ± 0.128 aA	1.993 ± 0.274 bA
	DOC (mg/kg)	512.895 ± 40.198 aA	420.473 ± 37.061 aB	550.535 ± 40.097 aA	469.265 ± 73.594 aA
	WSN (mg/kg)	86.341 ± 14.255 aAB	51.846 ± 7.982 bA	55.042 ± 3.030 bB	45.895 ± 6.954 bB
Jan.	pH	6.560 ± 0.233 bA	7.048 ± 0.066 aA	7.149 ± 0.029 aA	7.208 ± 0.034 aA
	NO_3-N (mg/kg)	14.267 ± 3.898 aB	10.476 ± 3.021 aB	12.220 ± 3.337 aB	21.702 ± 8.286 aAB
	NH_4-N (mg/kg)	82.743 ± 10.951 aA	41.587 ± 2.899 bB	63.613 ± 3.977 aB	70.176 ± 5.513 aB
	TP (mg/kg)	1351.600 ± 95.343 aB	626.625 ± 31.191 bA	676.500 ± 44.011 bB	719.650 ± 34.685 bA
	TN (%)	0.388 ± 0.030 aA	0.385 ± 0.048 aA	0.423 ± 0.051 aA	0.235 ± 0.025 bB
	TOC (mg/kg)	1.729 ± 0.136 bA	2.495 ± 0.213 aA	2.664 ± 0.160 aAB	1.785 ± 0.230 bA
	DOC (mg/kg)	279.43 ± 19.141 bC	400.582 ± 54.014 aB	243.487 ± 23.92 bC	231.237 ± 26.702 bB
	WSN (mg/kg)	41.707 ± 15.240 aB	34.08 ± 7.790 aA	24.345 ± 3.123 aB	33.693 ± 8.880 aB

Table S6. Potentially toxic element content in sediments of aquaculture ponds in the Jianghan Plain. Different lowercase letters indicate significant differences (*p*-value < 0.05) among different aquaculture patterns within the same aquaculture season. Different uppercase letters indicate significant differences (*p*-value < 0.05) among different aquaculture season within the same aquaculture patterns.

		FF	RS	SF	CF
Apr.	Zn (mg/kg)	190.35 ± 20.476 abA	146.975 ± 13.897 bA	201.675 ± 11.419 aA	173.825 ± 21.074 abA
	Pb (mg/kg)	29.450 ± 1.607 aA	19.175 ± 2.646 bAB	22.150 ± 2.168 bA	23.875 ± 1.578 abA
	Ni (mg/kg)	56.075 ± 0.600 aA	49.950 ± 1.627 bA	53.725 ± 1.423 abA	51.325 ± 1.517 bAB
	Mn (mg/kg)	581.975 ± 40.914 aA	377.650 ± 25.832 cA	510.400 ± 36.750 abA	439.075 ± 23.779 bcAB
	Cr (mg/kg)	121.225 ± 3.663 aA	115.625 ± 2.125 aA	109.525 ± 2.028 bA	109.65 ± 1.500 bB
	Cu (mg/kg)	72.150 ± 3.119 aBC	51.225 ± 1.958 bA	65.675 ± 2.837 aA	67.850 ± 1.902 aA
	As (mg/kg)	47.008 ± 5.345 aA	28.573 ± 4.431 bA	49.475 ± 6.199 aA	51.255 ± 6.015 aA
	Cd (mg/kg)	0.352 ± 0.016 aA	0.375 ± 0.008 aB	0.341 ± 0.025 aB	0.341 ± 0.032 aA
	Hg (mg/kg)	0.083 ± 0.017 aA	0.040 ± 0.001 bA	0.046 ± 0.004 bA	0.039 ± 0.003 bA
Jul.	Zn (mg/kg)	202.525 ± 20.420 aA	145.450 ± 16.129 aA	175.525 ± 20.468 aA	166.200 ± 22.153 aA
	Pb (mg/kg)	24.650 ± 2.780 aAB	22.900 ± 2.040 aA	20.700 ± 3.079 aA	24.375 ± 2.328 aA
	Ni (mg/kg)	53.975 ± 1.293 abAB	47.875 ± 1.412 bA	53.025 ± 2.847 abA	56.725 ± 4.478 aA
	Mn (mg/kg)	520.850 ± 39.052 aA	347.300 ± 21.611 bAB	529.825 ± 61.622 aA	477.725 ± 32.334 aA
	Cr (mg/kg)	109.500 ± 2.368 abB	104.250 ± 3.377 bB	106.625 ± 3.627 bAB	117.525 ± 3.917 aA
	Cu (mg/kg)	80.325 ± 4.177 aAB	53.125 ± 1.718 cA	65.125 ± 2.740 bA	71.650 ± 2.720 bA
	As (mg/kg)	45.752 ± 3.895 aA	40.134 ± 5.085 aA	44.174 ± 5.158 aA	40.468 ± 5.442 aAB
	Cd (mg/kg)	0.408 ± 0.020 aA	0.459 ± 0.018 aA	0.434 ± 0.020 aA	0.402 ± 0.028 aA
	Hg (mg/kg)	0.040 ± 0.011 aB	0.023 ± 0.003 aB	0.015 ± 0.002 aB	0.030 ± 0.013 aA
Oct.	Zn (mg/kg)	237.850 ± 16.294 aA	167.650 ± 11.057 bA	158.500 ± 11.064 bA	137.725 ± 11.920 bA
	Pb (mg/kg)	25.925 ± 3.213 aAB	20.300 ± 2.728 abAB	15.100 ± 3.088 bcA	10.950 ± 3.257 cB
	Ni (mg/kg)	57.025 ± 2.621 aA	47.425 ± 1.762 bA	49.975 ± 1.792 bA	48.825 ± 1.781 bAB
	Mn (mg/kg)	497.975 ± 34.236 aA	326.400 ± 17.956 bAB	436.525 ± 36.599 aA	464.600 ± 30.050 aA
	Cr (mg/kg)	110.650 ± 3.947 aB	97.250 ± 2.505 bB	100.850 ± 1.796 bB	103.225 ± 2.173 abBC
	Cu (mg/kg)	89.950 ± 6.036 aA	53.125 ± 1.582 cA	63.575 ± 3.072 bcA	65.550 ± 3.082 bA
	As (mg/kg)	34.860 ± 5.954 aAB	33.740 ± 3.739 aA	36.648 ± 4.063 aAB	36.133 ± 4.039 aAB
	Cd (mg/kg)	0.370 ± 0.023 aA	0.423 ± 0.020 aA	0.406 ± 0.023 aAB	0.384 ± 0.021 aA
	Hg (mg/kg)	0.036 ± 0.008 aB	0.028 ± 0.003 aB	0.038 ± 0.006 aA	0.034 ± 0.004 aA
Jan.	Zn (mg/kg)	177.300 ± 21.933 aA	142.825 ± 8.819 aA	171.975 ± 18.989 aA	166.625 ± 24.467 aA
	Pb (mg/kg)	19.400 ± 2.146 aB	15.775 ± 1.050 aB	15.575 ± 2.388 aA	17.800 ± 1.490 aA
	Ni (mg/kg)	49.975 ± 1.133 aB	46.150 ± 1.455 aA	51.225 ± 1.880 aA	47.700 ± 2.112 aB
	Mn (mg/kg)	385.950 ± 32.511 aB	287.575 ± 35.070 bB	403.675 ± 28.834 aA	379.275 ± 25.508 aB
	Cr (mg/kg)	99.725 ± 1.644 aC	100.625 ± 1.448 aB	102.800 ± 2.249 aAB	99.550 ± 1.210 aC
	Cu (mg/kg)	63.600 ± 3.652 aC	44.625 ± 1.360 cB	54.900 ± 2.898 bB	52.625 ± 3.465 bcB
	As (mg/kg)	26.815 ± 1.953 aB	12.342 ± 3.198 bB	26.346 ± 5.858 aB	29.244 ± 4.806 aB
	Cd (mg/kg)	0.352 ± 0.016 aA	0.375 ± 0.008 aB	0.344 ± 0.025 aB	0.338 ± 0.033 aA
	Hg (mg/kg)	0.083 ± 0.017 aA	0.040 ± 0.001 bA	0.045 ± 0.004 bA	0.042 ± 0.003 bA

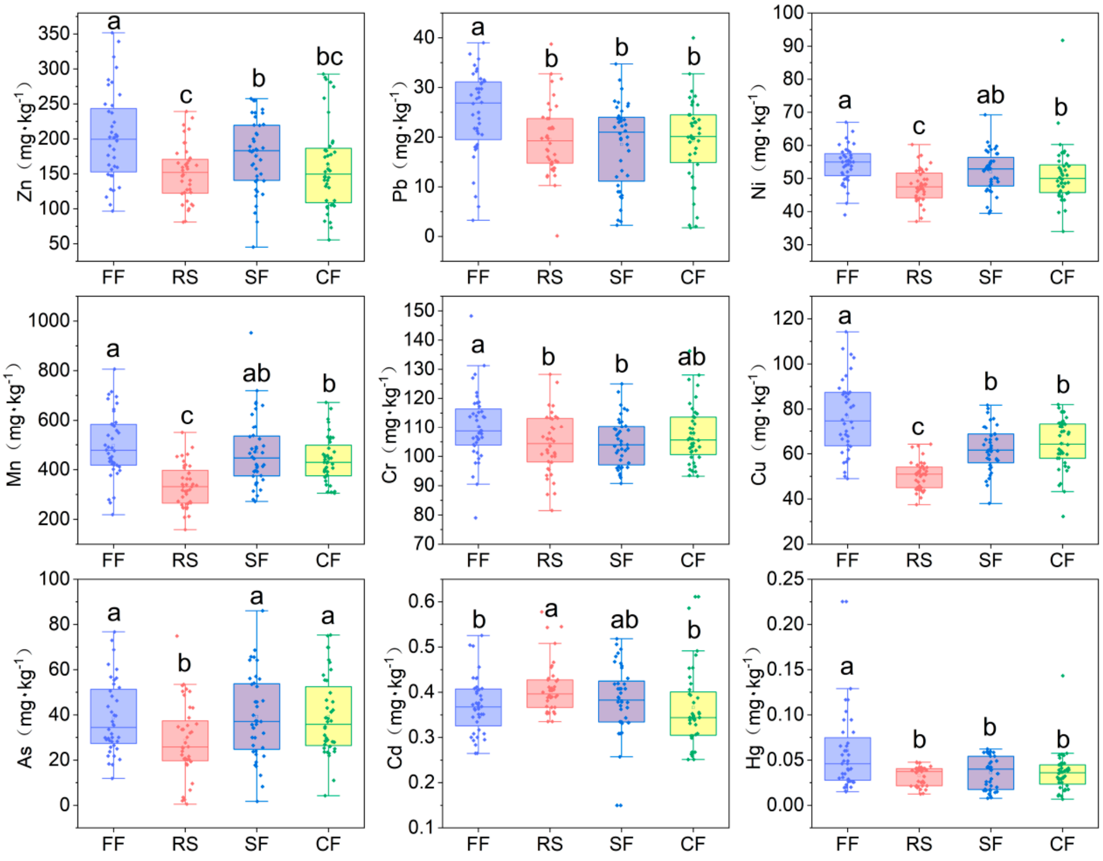

Fig. S1. Effects of different farming patterns on the contents of nine potentially toxic elements in aquaculture pond sediments. Different lowercase letters indicate significant differences at the 0.05 level.

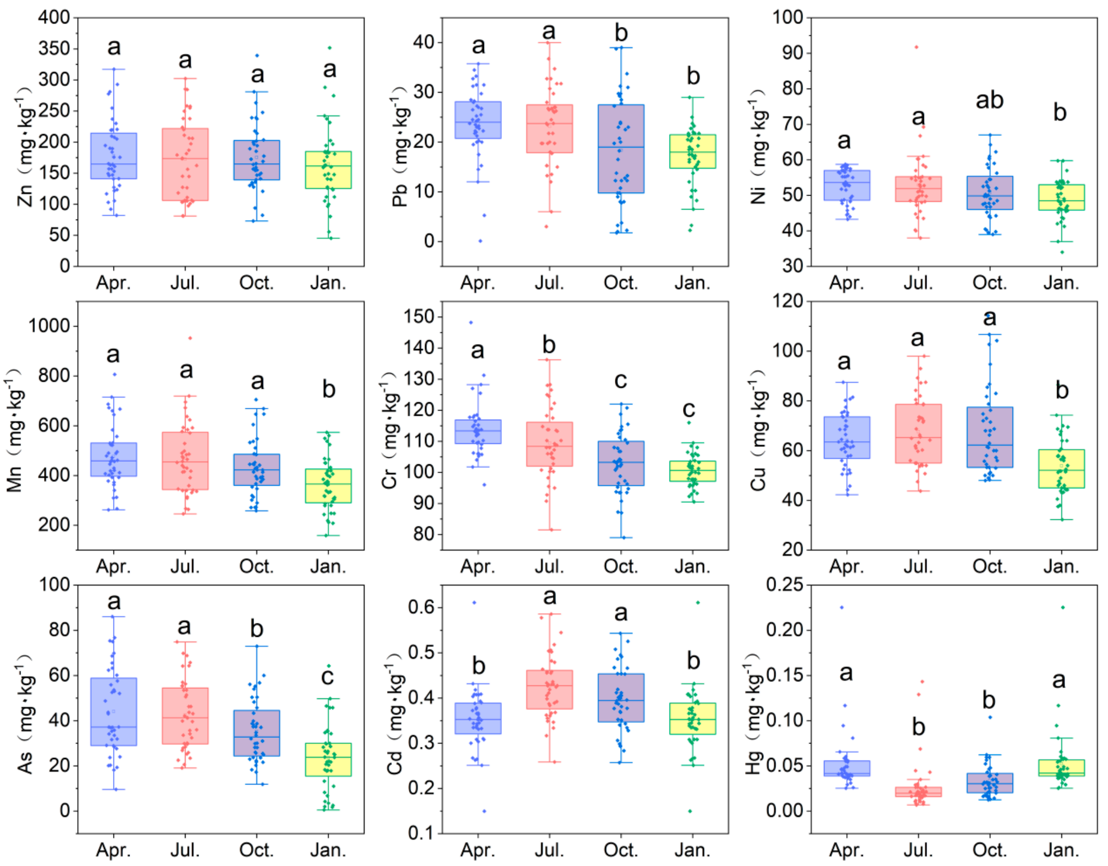

Fig. S2. Effects of different aquaculture seasons on the contents of nine potentially toxic elements in sediment samples from aquaculture ponds. Different lowercase letters indicate significant differences at the 0.05 level.

References

Hakanson, L. An ecological risk index for aquatic pollution-control - a sedimentological approach. Water Research, 1980, 14(8), 975-1001.
